# Homogeneous single-label cGMP detection platform for the functional study of nitric oxide-sensitive (soluble) guanylyl cyclases and cGMP-specific phosphodiesterases

**DOI:** 10.1038/s41598-020-74611-x

**Published:** 2020-10-15

**Authors:** Kari Kopra, Iraida Sharina, Emil Martin, Harri Härmä

**Affiliations:** 1 Department of Chemistry, Chemistry of Drug Development, University of Turku, Vatselankatu 2, 20500 Turku, Finland; 2 Division of Cardiology, Department of Internal Medicine, University of Texas Medical School At Houston, 1941 East Road, Houston, TX 77054 USA

**Keywords:** Enzymes, Biochemical assays

## Abstract

Cardiovascular diseases are the number one death worldwide. Nitric oxide (NO)—NO-sensitive (soluble) guanylyl cyclase (sGC)—cyclic guanosine monophosphate (cGMP) pathway regulates diverse set of important physiological functions, including maintenance of cardiovascular homeostasis. Resting and activated sGC enzyme converts guanosine triphosphate to an important second messenger cGMP. In addition to traditional NO generators, a number of sGC activators and stimulators are currently in clinical trials aiming to support or increase sGC activity in various pathological conditions. cGMP-specific phosphodiesterases (PDEs), which degrade cGMP to guanosine monophosphate, play key role in controlling the cGMP level and the strength or length of the cGMP-dependent cellular signaling. Thus, PDE inhibitors also have clear clinical applications. Here, we introduce a homogeneous quenching resonance energy transfer (QRET) for cGMP to monitor both sGC and PDE activities using high throughput screening adoptable method. We demonstrate that using cGMP-specific antibody, sGC or PDE activity and the effect of small molecules modulating their function can be studied with sub-picomole cGMP sensitivity. The results further indicate that the method is suitable for monitoring enzyme reactions also in complex biological cellular homogenates and mixture.

## Introduction

Cyclic 3´,5´-guanosine monophosphate (cGMP) is an important intracellular second messenger involved in various aspects of cell physiology and tissue homeostasis^1–3^. A wide spectrum of cellular and physiological functions in health and disease are affected by changes of intracellular cGMP levels. Vasodilation, inflammation, platelet function, solute balance, vascular regeneration, and neurotransmission are just few examples of these processes. The balance between the activity of guanylyl cyclases (GCs), which generate cGMP from guanosine triphosphate (GTP), and the activity of cGMP-specific phosphodiesterases (PDEs), which degrades cGMP into guanosine monophosphate (GMP), determines cellular cGMP concentration.

Mammals express seven single membrane-spanning isoforms of GCs, often referred as particulate GC or receptor guanylyl cyclase. Depending on the specific isoform and expressed tissue, these particulate GCs mediate the signal generated by natriuretic peptides, guanylins and heat-stable enterotoxin, light and calcium fluxes, CO_2_ and odors, and possibly even cold^4,5^. In addition to membrane-bound GC, cGMP is also generated by heterodimeric α1β1 or α2β1 cytosolic guanylyl cyclases GC-1 or GC-2, which are usually referred as soluble GC (sGC) or nitric oxide (NO)-sensitive GC, as the cGMP-forming activity is specifically and highly activated by gaseous signal messenger NO. sGC is regarded as the primary receptor of NO, as it mediates most of the known physiological functions of NO. These actions of GCs are opposed by PDEs, which catalyze the degradation of cGMP. Mammalian PDE superfamily contains 11 gene families (subtypes PDE1-PDE11), each having different affinities to either cGMP or cyclic adenosine monophosphate (cAMP)^6^. PDE5, 6, and 9 are specific for cGMP, whereas PDE1, 2, 3, 10, and 11 can hydrolyze both cAMP and cGMP.

There is clear evidence of specific cellular co-localization of cGMP-generating and cGMP-degrading enzymes. For example in cardiomyocytes, the paramembrane pool of natriuretic peptide activated GCs generate cGMP, which is controlled by PDE2, whereas the pool of cytosolic cGMP generated in response to NO is targeted mainly by PDE5^7–10^. Considering the role of cGMP in a plethora of physiologic processes, it is not surprising that both cGMP-generating and cGMP-degrading enzymes are targets for pharmacological intervention. Classical organic nitrates, e.g. nitroglycerine or isosorbides, have been used to manage angina and congestive heart failure long before it was determined that they generate NO^11^. However, the timeframe for nitrovasodilators use is limited, since patients develop tolerance^12^, while accompanying generation of reactive nitrogen intermediates affecting protein, lipids, and DNA, exacerbating the very condition that these drugs are intended to treat^13,14^. Last two decades we have witnessed the discovery and development of a number of NO-independent allosteric sGC regulators. Many of these regulators have entered into clinical trials or are already introduced in clinical practice^15–18^. It has already been shown that stimulators and activators of sGC and specific PDE inhibitors have clear therapeutic potential in various human disease conditions^15–19^. However, since cardiovascular disease is one of the leading cause of death worldwide, there is a continuous need and interest in identifying new and improving the existing drugs that modulate intracellular cGMP balance.

Even with the obvious need, the number of simple and efficient bioanalytical assays and drug screening methodologies to monitor cGMP concentration are limited. Most of the current methods for cGMP detection and quantification are based on direct cGMP concentration monitoring using heterogeneous enzyme-linked immunosorbent assays (ELISA) utilizing cGMP antibodies, radioimmunoassay (RIA) or HPLC-based cGMP detection^20–23^. Although sensitive, these methods contain various time- and labor-consuming separation steps. An indirect way to monitor sGC activity is to measure pyrophosphate (PP_i_) concentration, the secondary product formed in the reaction where cGMP is created from GTP^23,24^. Direct fluorometric and colorimetric assays for PP_i_ are commercially available, but they are less sensitive than heterogeneous methods, and suitable primarily for fully purified proteins. In addition, PP_i_ methods cannot be used to monitor PDE activity. None of these methods for cGMP meets all the requirements for an efficient high throughput screening (HTS) platform with a simple homogeneous protocol, short assay time and real-time readout, easy automation, and low costs. To meet these demands, cell-based assay for sGC activator screening have been developed^25,26^. The strategy utilizes cyclic nucleotide-gated channels and Ca^2+^-sensitive photoprotein as an indirect way to monitor small molecule induced cGMP production in real-time using specifically designed recombinant cell line. However, it does not allow for quantification of cGMP levels or measurement of PDE activity. Moreover, the protocols are somewhat laborious and time-consuming, which limits the use^25^. Energy transfer utilizing fusion protein biosensors, designed based on cGMP binding proteins, has also developed^26^. These assays utilize two fluorescent proteins, or donor enzyme and an acceptor fluorophore, and can be adapted to cGMP formation and degradation studies in vitro. However, the concept is more applicable for intracellular cGMP detection, where the fusion biosensor can be produced in cell of interest without purifications. Moreover, because of the indirect strategy of cGMP detection, the method has limited potential in quantifying cGMP levels.

We have previously introduced a homogeneous single lanthanide label (Ln(III)-chelate) utilizing technique called quenching resonance energy transfer (QRET)^27,28^. In QRET, the Ln(III)-chelate is conjugated to a small molecular weight ligand, creating a fluorescently labeled ligand, whose signal can be quenched in the presence of a soluble modulator. Receptor-ligand interaction creates a change in the local environment of Ln(III)-chelate, causing significant protection from the quencher, as compared to a much higher exposure of the ligand to the signal quencher in a non-bound state in solution. Therefore, upon binding of the Ln(III)-ligand to its receptor, a high fluorescence signal is observed. As in fluorescence polarization (FP), also applicable for homogeneous monitoring of cGMP forming and consumption activity^29,30^, only one label conjugation is needed for QRET, which simplifies assay optimization. However, unlike in FP performed with traditional organic fluorophores, QRET utilizes millisecond scale fluorescence lifetime of the Ln(III)-chelate, which is shown to improved sensitivity compared to FP in competitive assay^31,32^. In addition, wash-free homogeneous protocol ensures short assay time, while time-resolved fluorescence (TRF) detection provided a low background fluorescence even in biological milieu, as shown with e.g. with GTP and cAMP^33–36^.

Here, we report a homogeneous antibody-based method for cGMP detection. The method was applied to study sGC activity and its pharmacological regulators, and also the PDE activity and inhibition. The results demonstrate the efficacy, simplicity, and sensitivity of the homogeneous competitive cGMP detection platform, and indicate the wide applicability of the proposed technique. Method can also be easily adopted for HTS using either purified proteins or complex cellular homogenates.

## Results and discussion

**Figure 1:**
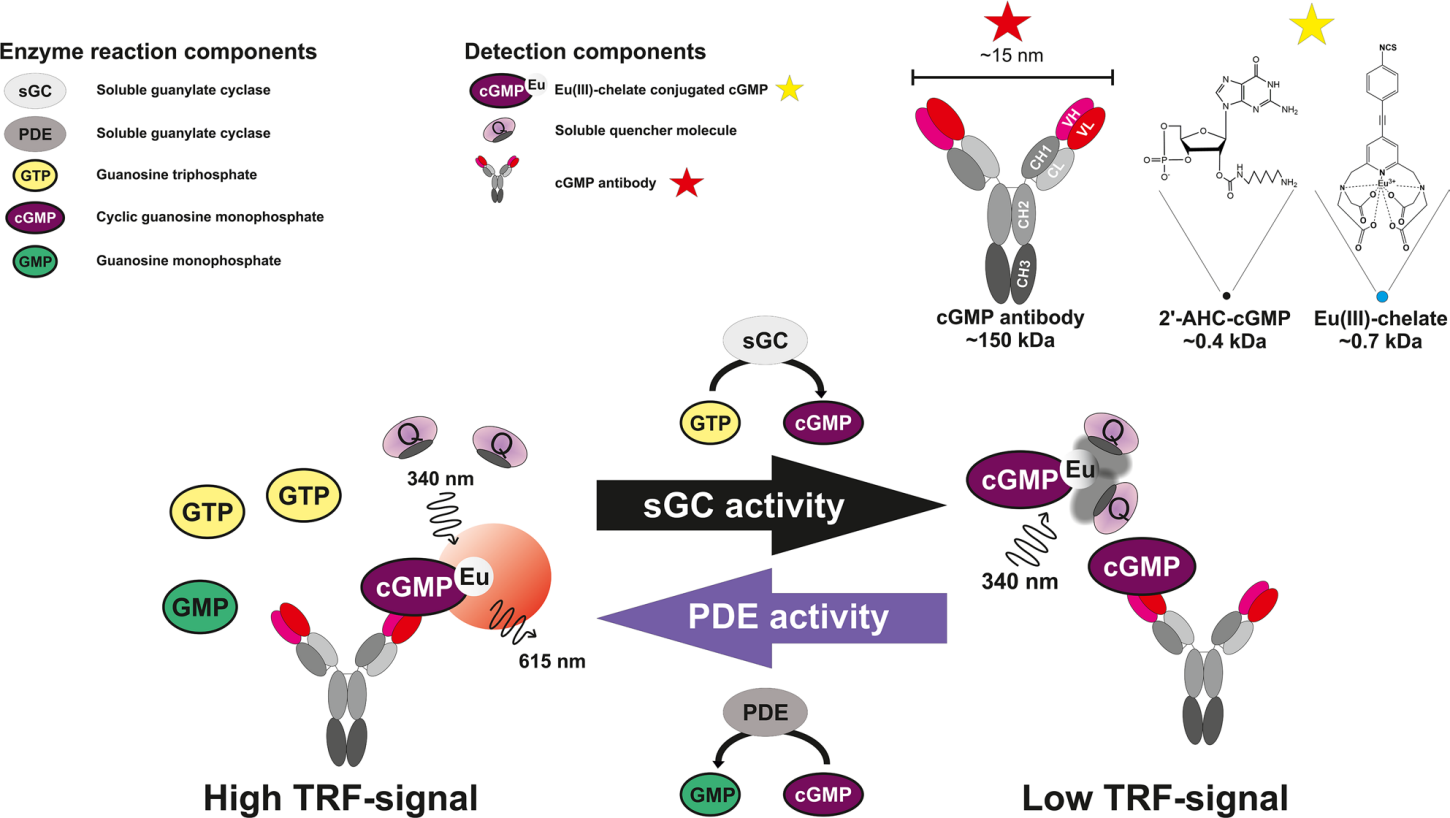
Principle of the homogeneous cGMP assay utilizing QRET detection. In the competitive QRET assay, high time-resolved fluorescence (TRF) signal is observed when no competing nucleotide is present and Eu(III)-cGMP is bound to the antibody protecting it from the soluble quencher. Increased cGMP concentration outcompetes the antibody bound Eu(III)-cGMP, exposing it to the soluble quencher and ultimately lowering the TRF-signal. In sGC assay, active sGC converts GTP to cGMP diminishing the observed TRF-signal. In PDE assay, the level of competing cGMP decreases, resulting in rebounding of Eu(III)-cGMP to the antibodies and increased TRF-signal is observed. As the assay principle is not demonstrated in scale, the protective effect creating true size difference between antibody (~ 150 kDa) and Eu(III)-cGMP (~ 1.5 kDa) is separately demonstrated.

We have previously described antibody-based nucleotide detection systems for GTP and cAMP^33,35,37^. In this report, we utilize the homogeneous single-label QRET principle to establish a HTS-compatible competitive cGMP detection method applicable for enzyme activity monitoring. The simplified principle of the method is presented in Fig. 1. In this assay, high cGMP concentration results in low TRF-signal since cGMP efficiently blocks Eu(III)-cGMP from binding to the cGMP-specific antibody, leaving the Eu(III)-cGMP in solution where it is quenched by the presence of modulator, and vice versa. The interaction with specific antibody creates locally protected environment for the significantly smaller size Eu(III)-cGMP, as in solution the efficient quenching is ensured by weak interactions between Eu(III)-cGMP and the soluble modulator^38^. Thus, the high cGMP concentration, produced by active sGC, can be monitored from low TRF-signal. Similarly, an inactive sGC or active cGMP-specific PDE produces high TRF-signal due to low cGMP concentration (Fig. 1).

**Figure 2:**
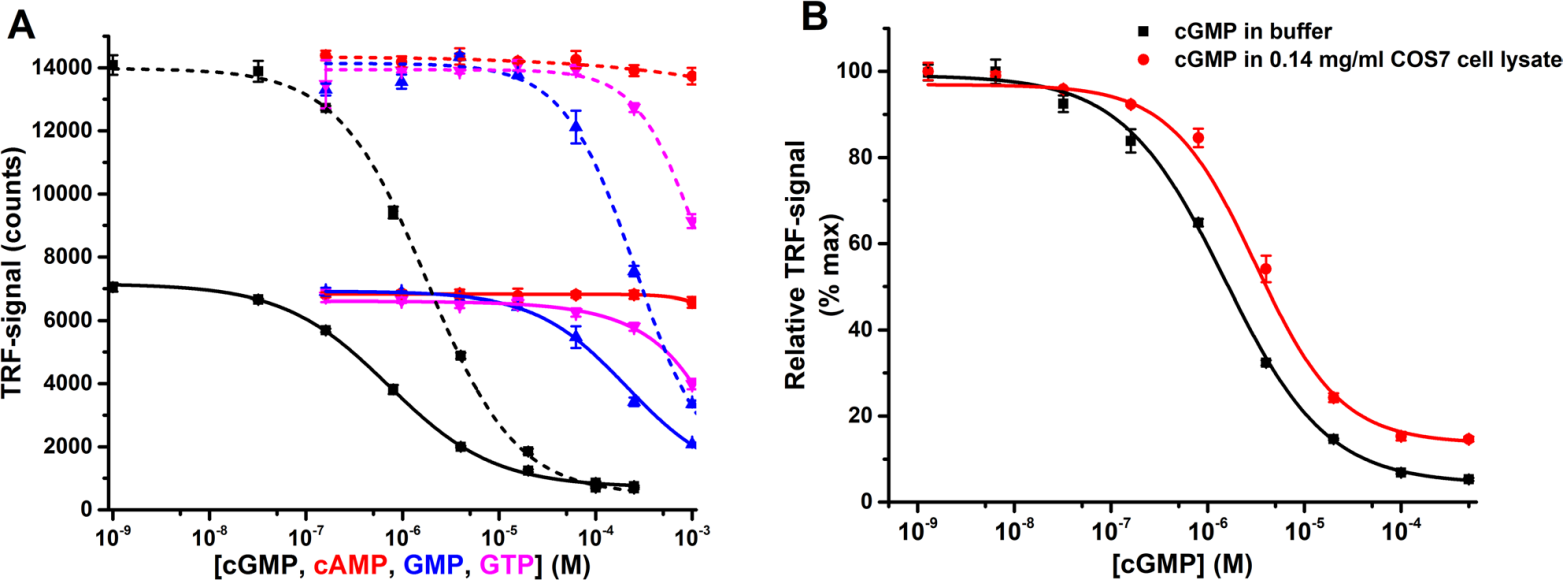
Optimization of the single-label QRET assay for homogeneous cGMP detection. (**A**) TRF-signals from antibody-bound Eu(III)-cGMP in the presence of different concentrations of cGMP (black), cAMP (red), GMP (blue), and GTP (magenta). Assay was performed using 1 nM Eu(III)-cGMP and 2 ng of antibody (solid line) or with 5 nM Eu(III)-cGMP and 4 ng of antibody (dashed line). (**B**) TRF-signal from 5 nM Eu(III)-cGMP and 4 ng of antibody monitored at the presence of different amounts of cGMP (0–500 µM) measured in buffer (black) or in COS7 lysate with a final protein concentrations of 0.14 mg/ml (red). Data represent the mean ± SD of three independent experiments performed in triplicates.

For the assay, we selected commercial monoclonal cGMP antibody from NewEast Biosciences. ELISA compatible antibodies are often directly QRET applicable, and to determine if this in the case in the designed cGMP detection platform, we tested it against different levels of cGMP and a panel of other nucleotides (Fig. 2A). Detection was performed using two detection setups to determine the effect in sensitivity, selectivity, and S/B ratio. With 1 nM Eu(III)-cGMP and 2 ng of antibody/well the EC_50_ value, limit of detection (LOD), and S/B ratio were 0.72 ± 0.07 µM, 33.6 nM, and 9.4, respectively (Fig. 2A). Similarly, with 5 nM Eu(III)-cGMP and 4 ng of antibody/well the EC_50_, LOD, and S/B ratio were 1.80 ± 0.22 µM, 104 nM, and 20.7, respectively (Fig. 2A). Determined LOD values signify that as little as 0.3–1.0 pmol of cGMP can be detected from a single well. Even the assay is homogeneous and competitive in nature, this sensitivity is similar or better to previously developed heterogeneous method utilizing direct cGMP detection^23,39^. The difference in assay time is even more drastic, as it is counted as minutes using the QRET-based cGMP detection, compared to hour with most of the other methods^23^. All measurements showed also good linearity and reproducibility. The linear range for cGMP detection was 0.032–20 µM and 0.032–100 µM, for each detection condition, respectively. As expected, slightly longer linear range was observed with higher antibody concentration. The linear range, where observed signal change is directly comparable to cGMP concentration, is always shorter in competitive compared to direct assay. Thus, this optimization step is crucial, as the cGMP binding curve in is sigmoidal in nature, and the length of the linear portion is a function of both antibody and Eu(III)-cGMP concentrations. Other nucleotides, cAMP, ATP, and ADP could not be recognized by the antibody (data not shown), and the specificity for cGMP was 200- and 1000-fold over GMP and GTP, respectively (Fig. 2A). Therefore, the linear range of cGMP detection was maintained when the assay was performed in the presence of 0.5 mM GTP (Fig. S1), indicating suitability for GC assays. In all assays, the maximal TRF-signal was observed after 5 min and thereafter the TRF-signal was found to stay stable over time.

The sensitivity and selectivity of the assay is in direct relation with the antibody affinity and specificity. Therefore, the K_d_ value for the used antibody was determined. Based on cGMP titration using fixed antibody concentration (4 ng/well) and five Eu(III)-cGMP concentrations (0.5–10 nM), the calculated K_d_ value for Eu(III)**-**cGMP binding to this antibody was 0.42 ± 0.05 µM (Fig. S2)^38,40^. This data suggest that the selected antibody is sufficient for cGMP monitoring, even it possess only high nanomolar affinity for Eu(III)**-**cGMP. Moreover, the used antibody performed equally well when the assay was performed in the presence of COS7 lysate (Fig. 2B). One clear advantage in QRET-based assays is that antibody can be displaced without additional labeling, as long as the presence of Ln(III)-chelate is tolerated. In case of cGMP, there is only single position expected to be tolerated for label conjugation, and this conjugation is not expected to interfere with cGMP recognition. Therefore the sensitivity of the assay may be further improved if better anti-cGMP antibodies or affinity agents becomes available.

Once the cGMP assay was optimized, we moved to test the platform using enzymes producing or consuming cGMP. In all assays, a cGMP standard curve was generated. The linear part of the cGMP standard curve, where relationship between TRF-signal and cGMP are comparable, was used to calculate the amount of cGMP generated/degraded in the reaction. This was also further used to determine the total and specific activities for the studied enzyme. We first determined if cGMP detection platform can be adopted to measure the cGMP-forming activity of sGC. Previous studies demonstrated that for resting sGC the K_m_ for GTP is ~ 220 µM, while for NO-activated sGC it is < 75 µM^41^. Therefore, 0.5 mM GTP, which does not interfere with assay’s sensitivity, can be expected to provide a robust cGMP-generating activity for resting or activated sGC. To optimize the enzyme assay conditions, cGMP production was measured at 37 °C using different amounts of sGC (0.04–276 ng/well) and different reaction times (10–60 min), both in resting conditions or in the presence of 100 µM DEA-NO, a spontaneous NO donor. The assay was performed using endpoint protocol in which the reaction was stopped by chelating the essential Mg^2+^ with EDTA, added in the detection mixture together with the used antibody, Eu(III)-cGMP, and soluble signal modulator (MT2). This optimization efforts allowed us to determine the range of sGC amounts and reaction times, when the activity of both resting and NO-stimulated sGC can be monitored fitting the linear range of cGMP-generating reaction (Fig. S3).

**Figure 3:**
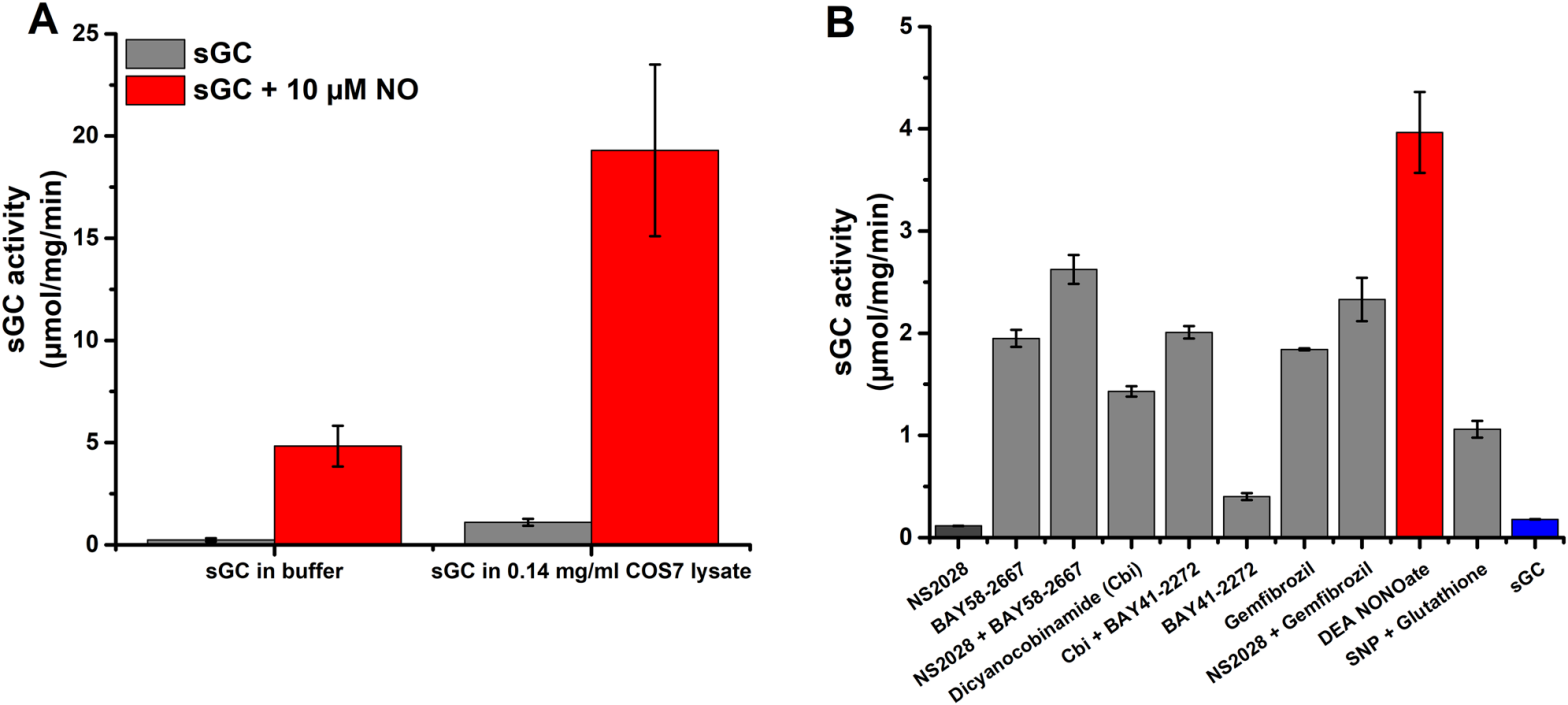
cGMP detection platform enables measurements of sGC activity and screening of sGC regulators. (**A**) sGC activity was determined in a 10 min reaction at 37 °C in the presence (red) or absence (grey) of 10 µM DEA-NO, using 5 ng sGC/well. The activity was measured in buffer or in the presence of 0.14 mg/ml COS7 lysates. The amount of generated cGMP was calculated from cGMP standard curve obtained as described in “Methods”. Data are mean ± SD of four independent experiments performed in triplicate. (**B**) A panel of sGC inhibitors, stimulators, activators or indicated combinations were tested and compared to the activity of resting (blue) or 10 µM DEA-NO-treated (red) sGC in a 10 min reaction at 37 °C, using 5 ng sGC/well. All regulators, except NS2028 (dark gray), showed decreased TRF-signal due to increased cGMP-generation activity of stimulated sGC. The amount of generated cGMP was calculated from the change in TRF-signal in comparison to the linear part of cGMP standard. Data represent the mean ± SD of three independent experiments performed in triplicates.

To validate the sGC assay further, we used the selected 10 min incubation at 37 °C, and determined the specific activity of purified sGC. As shown in Fig. 3A, resting sGC activity was estimated at 0.23 ± 0.09 µmol/min/mg, which is slightly higher than the values reported using direct [α32P]-GTP → [α32P]-cGMP method^24,42^. On the other hand, NO-stimulated activity was estimated at 4.8 ± 1.0 µmol/min/mg (Fig. 3A), corroborating well with the previously reported values^24,42^. Although the reason for elevated activity for the resting sGC is not entirely clear, it results in an apparently lower fold of NO-dependent sGC stimulation.

We also measured sGC activity in the presence of cell lysate to evaluate any possible interference that a biological mixture may have on the assay. COS7 cells lack sGC activity and are well suited for this purpose^43^. In this case, the cGMP detection platform also exhibited strong activation of sGC by DEA-NO, indicating that the functionality of the detection system is not compromised even in complex biological mixture (Fig. 3A). In the presence of COS7 lysate, the average calculated activity for resting sGC was 1.0 ± 0.2 µmole/mg/min, while for NO-stimulated sGC is was 19.3 ± 4.2 µmole/mg/min (Fig. 3A). The higher values observed in the presence of COS7 lysates corroborate previously reported findings that COS7 lysates enhance sGC activity^44^. It has been proposed that this elevated sGC activity is most likely due to the effect of 70 kDa heat shock protein (HSP70)^44^. Altogether, these data clearly demonstrates the versatility of cGMP detection platform and its usefulness in monitoring cGMP-forming activity.

Next, we studied the robustness and reproducibility of the assay by monitoring 24 replicate reactions under resting or stimulated conditions. 100 µM gemfibrozil or 10 µM DEA-NO were used to activate sGC, and assays were performed with different enzyme preparations and measured at various time points (Fig. S4). Under the selected experimental conditions, we observed approximately 20- and 50-fold increase for cGMP in response to given concentrations of gemfibrozil and NO, as compared to untreated sGC. The assay exhibited good reproducibility, as the Z’-factor calculated from resting or gemfibrozil activated reactions after 10 and 60 min were 0.69 and 0.78, respectively (Fig. S4). Similarly, NO activation produced Z-factors of 0.86 and 0.83 (Fig. S4). The average Z-factor from gemfibrozil and NO activated reactions monitored at three consecutive assays using 24 reactions were 0.74 and 0.83 with the respective average S/B ratios of 3.2 and 5.1.

After confirming method’s reproducibility and potential suitability for HTS purposes, we validated the cGMP-detection platform by testing a number of known sGC modulators or their combinations. sGC activators and stimulators are highly attractive drug molecules for treatments of heart and vascular diseases, and thus novel molecules are of interest. First, we assayed the activity of 5 ng sGC in response to a number of known sGC modulators or their combinations (Fig. 3B). This panel included sGC stimulators YC-1, riociguat, BAY41-2272, heme-independent sGC activators BAY58-2667, and the fibrate drug gemfibrozil, which was previously reported to have sGC-activating properties^42^. The effect of cobinamide (Cbi), a vitamin B12 precursor that has been reported to target the catalytic region of sGC^24^, was also tested. At the selected saturating concentrations, all tested stimulators and activators showed clear sGC activity increase over the resting sGC activity (Fig. 3B), corroborating previous findings. As expected, DEA-NO exhibited the largest increase in sGC activity. NS2028, sGC inhibitor and heme oxidizer, was used as a negative control and it showed no significant effect on the activity of non-stimulated sGC. On the other hand, NS2028 increased the activation when assayed together with such compounds as BAY58-2667 or gemfibrozil, both of which were shown to preferentially activate heme-free sGC or sGC with oxidized heme^42,45^. We also analyzed the dose-dependent effect of selected sGC regulators, YC-1, riociguat, and DEA-NO. All of these compounds showed concentration dependent sGC activation, with a calculated EC_50_ values of 5.7 ± 1.0 µM, 0.26 ± 0.1 µM, and 0.93 ± 0.1 µM for YC-1, riociguat, and DEA-NO, respectively (Fig. S5). The monitored EC_50_ values are similar to previously reported values, considering that there is a large variance of the reported values based on the origin of sGC and the source of NO^46–49^. These assays highlights the assay suitability for sGC modulator testing.

We have previously shown that the QRET detection can be used to monitor reaction kinetics in real-time^36,38^. Thus we modified the detection scheme and tested if the sGC reaction could also be monitored. In the real-time protocol, sGC enzyme and detection components were added before the reaction was initiated with GTP or GTP/DEA-NO. Compared to the endpoint protocol, the calculated resting sGC activity was approximately tenfold lower (Fig. S6), while NO-stimulated sGC was significantly compromised, even the maximal S/B ratio was preserved (data not shown). We speculate that the diminished response to NO stimulus could be due to the presence of 0.01% Triton X-100, a strong nonionic detergent used to stabilize the QRET signals and to prevent non-specific effects during incubation. Therefore, it is expected that a progressive loss of heme occurs during the real-time assay. It has been previously demonstrated that even a less potent detergent, Tween 20, causes a significant loss of sGC heme and weakens the NO stimulation of sGC^50^. Another possible contribution is the relative slow kinetics of the detection system with the used antibody. As mentioned above, maximal TRF-signal develops after 5 min, and even in real-time assay the complex is ready formed, Eu(III)-cGMP displacement needs to be occurred. Thus, the assay may be insufficient to capture a representative portion of cGMP generates by the high cGMP output state of sGC, which is established after NO stimulation. Although these data indicate lack of suitability for quantitative kinetic measurement of cGMP level, it may still be used when only a qualitative result is needed. The real-time assay may also be applicable for measurements of particulate GC, which are less sensitive to Triton X-100 and have a lower cGMP-forming activity.

After proving the method suitability for cGMP production monitoring, we next tested if cGMP consumption could also be monitored. Mammalian PDE superfamily contains 11 genes, which participate cell signaling by controlling the cAMP and/or cGMP levels^51^. Three of these members are dedicated solely to the reaction of converting cGMP into GMP. Since cGMP antibody have more than 200-fold specificity to cGMP over GMP (Fig. 2A), we predicted that PDE activity could also be monitored using the developed cGMP assay.

**Figure 4:**
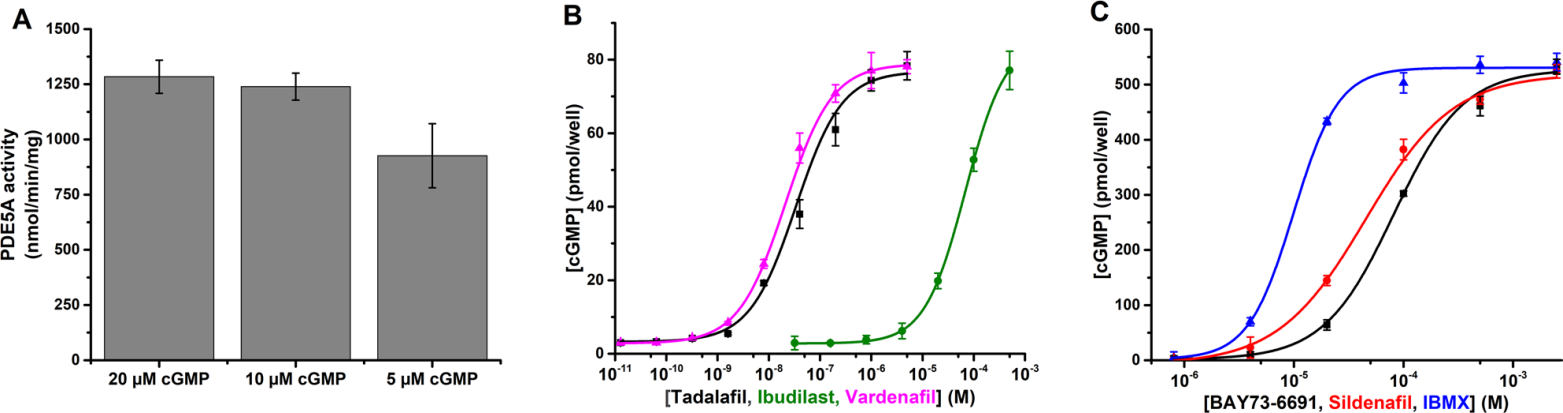
PDE activity inhibition can be monitored using cGMP detection platform. (**A**) Specific activity of PDE5A was determined in a four point assay using 0.5–5 ng/well of PDE5A. Using 10 min reaction time at room temperature, we determined similar PDE activity with all three cGMP concentrations (5–20 µM). (**B**) Dose-dependent inhibitions of PDE5A by tadalafil (black), ibudilast (olive), and vardenafil (magenta) were assayed with 75 pmol/well of cGMP and 15 ng/well of PDE5A in a 60 min reaction at RT. The final change in TRF-signal was used to calculate the amount of consumed cGMP based on cGMP standard. (**C**) cGMP (500 pmol/well) hydrolysis, in mouse brain homogenate (0.1 mg/ml) and in the presence of indicated concentrations of PDE9 inhibitor BAY73-6691 (black), PDE5 inhibitor sildenafil (red), and broad specificity inhibitor IBMX (blue), were studied in a 15 min reaction at 37 °C. All inhibitors showed PDE activity inhibition at micromolar level. Data represent the mean ± SD of three independent experiments performed in triplicates.

First, we monitored the changes in cGMP levels in response to different amounts of PDE5A enzyme and different starting cGMP concentrations (Fig. S7). Using a 60 min reaction time, we observed clear PDE5A and cGMP concentration dependence. To calculate specific activity of PDE5A, the reaction time was then limited to 10 min and cGMP consumption to 15% of the total amount of substrate (Fig. 4A). In assays performed at room temperature with 10 and 20 µM cGMP, a specific activity of 1284 ± 75 and 1239 ± 61 nmol/min/min were monitored, respectively. This corroborates well with the specific value of 1940 nmol/min/mg obtained at 30 °C, as provided by the PDE5A manufacturer. The activity observed with 5 µM cGMP was already slightly lower, 926 ± 145 nmol/min/min, which was expected since PDE5A has a K_m_ for cGMP of approximately 2 µM^52^. While these data demonstrate that QRET detection platform can be used to evaluate PDE5A activity, some limitations should be considered. For cGMP degradation, the measurable TRF-signal is initially low and the signal develops as cGMP is being degraded, resulting in less optimal S/B ratio for PDE specific activity determination. This is mainly due to the fact that cGMP concentration at the beginning of the reaction is not high enough for complete displacement of Eu(III)-cGMP and because not all cGMP substrate can be consumed to retain linearity. These restrictions require higher MT2 concentration to lower the TRF-signal levels, which improved the S/B ratio. Also Eu(III)-cGMP might have a potential to bind PDE, and thus assays were performed only using endpoint protocol.

We evaluated the effect of three PDE5A inhibitors, tadalafil, ibudilast, and vardenafil, using 60 min reaction time and selected cGMP (7.5 µM) and PDE5A (15 ng) concentrations (Fig. 4B). The observed IC_50_ values for tadalafil, ibudilast, and vardenafil were 33.7 ± 11 nM, 68.7 ± 0.4 µM, and 21.0 ± 2.3 nM, respectively, which are similar to those reported previously^53,54^. Even though PDE5A is not expected to reach maximal activity in this substrate consuming assay, these results demonstrates that the developed cGMP detection platform can be used for purified PDE enzyme, and especially to study PDE inhibitors.

The function of several PDEs has been linked to Alzheimer's disease and PDE inhibition is being considered as a potential therapeutic strategy for this disease^55^. Therefore, we applied the cGMP detection platform to probe PDE activity in complex mouse brain tissue homogenate, which contains a cocktail of different PDEs with different properties. Therefore, we selected a broad-spectrum PDE inhibitor (IBMX), a PDE9-specific (BAY73-6691), and a PDE5-specific (sildenafil) inhibitor for testing^55^. Since brain homogenate contains a number of cGMP-degrading PDEs, such as PDE1, 2, 5, 9, 10, and 11^56,57^, the assay was performed at 37 °C with 50 µM concentration of cGMP and 3 µM MT2 quencher, to retain linearity (Fig. 4C). In these conditions, the apparent IC_50_ values for IBMX, BAY73-6691, and sildenafil were 9.9 ± 0.6, 81.3 ± 4.8, and 45.2 ± 6.8 µM, respectively. The IC_50_ obtained for pan-specific IBMX is similar to previously reported values^58^. However, IC_50_ values for PDE5A-specific sildenafil and PDE9-specific BAY73-6691 were higher than expected, and reflects the presence of several different cGMP-specific PDEs, which can be efficiently inhibited only at high nonselective concentration of these inhibitors. Presented data indicate that the developed QRET-based cGMP detection platform can be adapted for measurement of PDE activity and screening of potentially new PDE regulators. However, one must keep in mind that the dynamic range of the QRET-based assay for competitive detection of cGMP is not as wide in cGMP consumption assay as in cGMP synthesis assay. This fact must be considered during the competitive QRET-based cGMP assay optimization, which is more demanding on concentration compared to traditional direct cGMP assays as RIA, having longer linear measurement area.

## Conclusions

In this work, we have developed and introduced a homogeneous cGMP detection platform and proved its functionality by monitoring sGC and PDE activity in the presence or absence of their activity modulators. The assay showed equal sensitivity and improved simplicity compared to current laborious heterogeneous methods, and in contrast to those, the readout is performed in minutes. The described assay for cGMP detection is HTS-compatible and potentially enables efficient screening for small molecule modulators not only for sGC and cGMP-specific PDE, but also potentially for membrane-bound GCs. These particulate GCs are not NO activated, and potentially may be more easily adapted to real-time monitoring of enzymatic reactions.

## Methods

### Materials and instrumentation

GTP, GMP, cGMP, cAMP, ATP, ADP, and γ-globulins from bovine blood were purchased from Sigma-Aldrich (St. Louis, MO, USA). Full-length sGC was purified from Sf9 cells as described previously^59^. COS7 cells were cultured in Dulbecco Modified Eagle Medium (DMEM) media supplemented with 10% fetal bovine serum and penicillin–streptomycin mix all purchased from Thermo Fisher Scientific (Gibco, Thermo Fisher Scientific, UK). COS7 lysates were prepared by sonication in 50 mM HEPES buffer pH 7.4. High-speed supernatant (6.8 mg/ml) were prepared by 100,000×*g* centrifugation. High-speed supernatants from the mouse brain homogenates (21 mg/ml) were prepared by using a Dounce homogenizer and subsequent centrifugation at 100,000xg. Recombinant human PDE5A (537-end) was from SignalChem (Richmont, Canada), and the batch specific reported activity monitored at 30 °C was 1940 nmol/min/min. The 2′-O-(6-aminohexylcarbamoyl)guanosine-3′,5′-cyclic monophosphate (2′-AHC-cGMP), used to prepare Eu(III)-cGMP, was purchased from BIOLOG Life Science Institute (Bremen, Germany). Heptadentate Eu(III)-chelate, ITC-TEKES-Eu(III), and the soluble quencher molecule, MT2, were obtained from QRET Technologies (Turku, Finland). Eu(III)-chelate conjugation to cGMP was performed as described previously with GTP^36^. Used cGMP and Eu(III)-chelate are depicted in Fig. 1. Monoclonal cGMP-specific antibody was purchased from NewEast Biosciences (Malvern, PA, USA). PDE and sGC activity modulators, diethylamine NONOate (DEA-NO), riociguat, 3-(5′-hydroxymethyl-2′-furyl)-1-benzyl indazole (YC-1), 8-bromo-4H-2,5-dioxa-3,9b-diaza-cyclopenta[a]naphthalen-1-one (NS2028), BAY58-2667, BAY41-2272, dicyanocobinamide (Cbi), gemfibrozil, sodium nitroprusside (SNP), glutathione, tadalafil, ibudilast, vardenafil, BAY73-6691, sildenafil, and 3-isobutyl-1-methylxanthine (IBMX) were from Sigma-Aldrich, Merck Life Sciences (San Diego, CA, USA), and Cayman Chemicals (Ann Arbor, MI, USA). White Corning round bottom low volume 384-well plates (4513) were used in all biochemical assays (Corning, Kennebunk, ME, USA). All other reagents including basic buffer components and analytical-grade solvents were from Sigma-Aldrich.

The Eu(III)-cGMP purification was carried out using reversed-phase adsorption chromatography, Dionex ultimate 3000 LC system from Thermo Fischer Scientific, Dionex (Sunnyvale, CA, USA), and Ascentis RP-amide C18 column from Sigma-Aldrich, Supelco Analytical (St. Louis, MO, USA)^36^. TRF-signals were measured at 615 nm, using 340 nm excitation wavelength (800 µs delay and 400 µs window), using a standard microtiter plate reader developed by Labrox Ltd. (Turku, Finland), Victor 1420 multilabel counter from PerkinElmer Life and Analytical Sciences, PerkinElmer Wallac (Turku, Finland) or Spark multimode reader from Tecan (Männedorf, Switzerland).

### Homogeneous cGMP detection functionality and optimization

All biochemical assays were performed in a 10 µL final volume using triplicate reactions and three individual experiments unless otherwise indicated. The non-enzymatic and real-time cGMP assays were performed in assay buffer 1 (20 mM HEPES, pH 7.5, 1 mM MgCl_2_, 0.005% γ-globulins, 0.01% Triton X-100), enzymatic end-point assays in assay buffer 2 (assay buffer 1 without 0.01% Triton X-100), and detection components (Eu(III)-GTP, antibody, and MT2) were added in the stop buffer (assay buffer 1 supplemented with 10 mM EDTA instead of 1 mM MgCl_2_). In all buffers, sGMP was equally detected. Buffers for end-point assay were optimized to avoid the used of Triton X-100 during the enzymatic sGC reaction, avoiding the progressive loss of heme, and by using the EDTA in stop buffer to chelate the enzyme essential MgCl_2_.

After initial assays, cGMP detection protocol was tested in cGMP titration with two antibody (2 ng/well and 4 ng/well) and two Eu(III)-cGMP (1 nM and 5 nM) concentrations. Titrated cGMP (0–250 µM) was added in 5 µL volume and detection solution containing Eu(III)-cGMP, cGMP antibody, and MT2 (1.2 µM or 2.2 µM) in 5 µL volume. Similar titrations were performed with other nucleotides (cAMP, GTP, ATP, ADP, and GMP). cGMP was also titrated in the presence of 0.5 mM GTP. In all of these assays, background controls were performed with six repeats and TRF-signals were monitored multiple times between 5 to 90 min to determine the TRF-signal stability.

cGMP binding affinity to the used cGMP-specific antibody was studied performing cGMP titration (0–500 µM) with five Eu(III)-cGMP concentrations (0.5–10 nM). cGMP was added in 5 µL volume and incubated with detection solution containing fixed antibody concentration (4 ng/well). Assays were performed with Eu(III)-cGMP concentration dependent individually fixed MT2 (1.2–3.2 µM) quencher concentration. TRF-signals were measured and the Eu(III)-cGMP equilibrium dissociation constant (K_d_) for the cGMP-specific antibody was determined using linearized Cheng-Prusoff Equation^59^.

Interference from biological matrix was evaluated by performing cGMP (0–500 µM) titration in assay buffer 2 or COS7 cell lysate (0.14 mg/ml protein). cGMP dilutions were mixed with buffer or diluted lysate in 5 µL volume before the addition of equal volume of detection solution and the TRF-signals measurement performed as previously.

### sGC activity assay

Optimized cGMP detection platform was applied to all enzymatic assays. For non-stimulated conditions, purified sGC enzyme (0.04–276 ng/well) was incubated with or without 0.5 mM GTP in the assay buffer 2 for 10 to 45 min at 37 °C in 5 µL volume. The reaction was stopped by adding 5 µL of detection solution (0.4 µg/ml cGMP antibody, 5 nM Eu(III)-cGMP, and 2.2 µM MT2) containing EDTA. For NO stimulated sGC activity, the same reactions were performed in the presence of 10 or 100 µM DEA-NO added with GTP. DEA-NO was always prepared from a 100 mM stock solution in 10 mM NaOH, which was stored at − 80 °C. TRF-signals were always monitored multiple times between 5 to 90 min to determine the TRF-signal stability.

In assays where dose-dependent effect of three sGC activators, DEA-NO, YC-1, and riociguat, were tested, the compounds (0–100 µM) were incubated with 2 ng sGC. Reactions were initiated by adding 0.5 mM GTP to reach 5 µL volume and incubated for 30 min at 37 °C. Reactions were stopped by adding 5 µL of detection solution and TRF-signals were recorded. To evaluate the linearity of the cGMP production by sGC, 3 or 12 ng of sGC were used for resting conditions and 0.5 ng for 100 µM DEA-NO activated sGC. Assays were performed in separate plates and reactions were stopped at indicated time points (5–60 min), before TRF-signal monitoring as above.

For assays containing fixed concentration of sGC regulators, 5 ng of sGC was incubated with one, or the indicated combination of following molecules: 10 µM NS2028, 100 nM BAY58-2667, 100 µM gemfibrozil, 10 µM BAY41-2272, 100 µM Cbi, 10 µM DEA-NO or SNP with glutathione (100 µM each). 5 µL reactions containing 0.5 mM GTP were incubated for 10 min at 37 °C and stopped by adding the detection components. TRF-signals were recorded as described above.

Cell lysate interferences were evaluated in an endpoint assay with different amounts of sGC (0.008–166.7 ng) and 0.07 or 0.14 mg/ml of COS7 cell lysate. sGC/lysate mixture was supplemented with 0.5 mM GTP in the presence or absence of 10 µM DEA-NO, and 5 µL reaction was incubated for 10 min at 37 °C. Detection components were thereafter added and TRF-signals were monitored as above.

Reproducibility and HTS compatibility were determined by running Z’-factor assay using 24 sGC reactions with or without activity modulation in three consecutive days. Assays were performed with fixed sGC concentration (5 ng/well) and sGC activation was performed using gemfibrozil (100 µM) or DEA NONOate (10 µM). All assays were initiated by the addition of 0.5 mM GTP to obtain a final 5 µL reaction volume, 37 °C for 10 min. Detection solution was added and TRF-signals were monitored as previously. Z’-factors were calculated using sGC basal activity as negative and activated reactions (NO or gemfibrozil) as positive controls.

For the real-time detection of sGC activity, all components were added in the assay buffer 1, containing both MgCl_2_ and Triton X-100. sGC and detection components were first added to obtain 8 µL volume and the reaction was initiated with 2 µL of GTP (0.5 mM) or GTP/DEA-NO (100 µM). TRF-signals from triplicate reactions were recorded at 37 °C every 5 min for 45 min.

In parallel to all assays, changes in TRF-signal in response to different concentrations of exogenously added cGMP were recorded to generate a cGMP standard curve. The cGMP standard was created using 1:3 dilution series to maximize the linear part of the standard, which was used to calculate the amount of cGMP generated by sGC during the reaction. Only at the linear area, the TRF-signal relation to cGMP concentration can be determined.

### cGMP-specific PDE activity assay

PDE activity was studied by performing PDE5A titration in the presence of 5, 10 or 20 µM of cGMP as a starting concentration. PDE5A (0–63 ng) and cGMP were both added in 4 µL volume and reaction was incubated for 60 min at RT. cGMP conversion to GMP was detected with similar detection solution as in sGC assay, but with higher 3.5 µM MT2 concentration. Detection components were added in 2 µL and TRF-signal was recorded as previously. For activity calculations, 0.5–5 ng/well of PDE5A was assayed in a 10 min reaction using 5, 10 or 20 µM cGMP substrate. For testing the dose-dependent effect of PDE5A inhibitors, different concentrations of tadalafil (0–5 µM), ibudilast (0–500 µM), and vardenafil (0–5 µM) were added to the 5 µL reaction containing PDE5A (15 ng) and cGMP (75 pmol) and incubated for 60 min at RT. Dimethyl sulfoxide (DMSO) used to dissolve inhibitors was kept at 1% in all assays. Detection solution was added in 5 µL and TRF-signals were monitored as previously.

PDE inhibitors with varying specificity profile, BAY73-6691, sildenafil, and IBMX (0–500 µM), were assayed in a high-speed supernatants of mouse brain homogenates. Reactions were performed in 5 µL volume in the presence of 0.1 mg/ml of homogenate and 50 µM cGMP. After 15 min incubation at 37 °C, detection solution (0.4 µg/ml cGMP antibody, 5 nM Eu(III)-cGMP, and 3.0 µM MT2) was added and TRF-signals were recorded.

For each cGMP degradation assay, the relationship between TRF-signal and different concentrations of exogenously added cGMP. By matching the TRF-signals to the linear part of the cGMP standard curve, consumed amount of cGMP during the PDE reaction was determined.

All experimental procedures related to animals were carried in accordance to approved safety protocols at UTHealth McGovern School of Medicine. All animal manipulations were approved by the Animal Welfare Committee of UTHealth, and meet all standards mandated by the Animal Welfare Act and the Public Health Service Policy on Humane Care and Use of Laboratory Animals.

### Data analysis

In all assays, the signal-to-background ratio (S/B) was calculated as µ_max_/µ_min_, coefficient of variation (CV%) (σ/µ)*100, and Z’-factor (1-[(3σ_max_ + 3σ_min_)/(│µ_max_-µ_min_│)]). The limit of detection (LOD) was defined as µ_min_ + 3σ. The K_d_ value was calculated using the linearized Cheng–Prusoff equation, IC_50_ = (([K_i_]/K_d_) x [C]) + K_i_^40^. The K_i_ value in the assay was calculated using the Cheng–Prusoff equation, EC_50_/(1 + [C]/K_d_). In the formulas µ is the mean value, and σ is the standard deviation (SD), IC_50_ is the half maximal inhibitory concentration, EC_50_ is the half maximal effector concentration, C is the Eu(III)-cGMP concentration, K_i_ is the inhibitory constant, and K_d_ is the dissociation constant. Specific activity of sGC or PDE5A was calculated by comparing TRF-signal data to the linear part of the cGMP standard curve, which was always monitored in parallel to studied reaction using the same assay conditions. Calculations were performed using equation, n (mol)/t (min)/m (mg), in which n refers the amount of cGMP produced or consumed, t is the assay time, and m is the amount of sGC or PDE enzyme used to perform the reaction. Data were analyzed and figures were drawn using Origin 8 software (OriginLab, Northampton, MA, USA)*.* All representative figures from the end-point assays were drawn from the data recorded 10 to 15 min after detection solution addition.

## Supplementary information

Supplementary file1
