# Supplementary material for: Homogeneous single-label cGMP detection platform for the functional study of nitric oxide-sensitive (soluble) guanylyl cyclases and cGMP-specific phosphodiesterases
Source: Sci Rep. 2020 Oct 15;10:17469. doi: 10.1038/s41598-020-74611-x (PMC7562898; doi:10.1038/s41598-020-74611-x)
Supplement: Supplementary file 1 — Supplementary file1 [file 41598_2020_74611_MOESM1_ESM.docx]

**Homogeneous single-label cGMP detection platform for the functional study of nitric oxide-sensitive (soluble) guanylyl cyclases and cGMP-specific phosphodiesterases**

Kari Kopra,^1,*^ Iraida Sharina,^2^ Emil Martin,^2^ and Harri Härmä^1^

^1^ Department of Chemistry, University of Turku, Finland

^2^ Department of Internal Medicine, University of Texas Medical School at Houston, USA

Corresponding Author: [kari.kopra@utu.fi](mailto:kari.kopra@utu.fi)

**Supplemental Information**

**Table of contents**

**Supplemental results**

**Figure S1.** Linearity of the cGMP detection

**Figure S2.** Eu(III)-cGMP binding affinity of the cGMP antibody

**Figure S3.** Linearity of the sGC’s cGMP-generating activity

**Figure S4.** Reproducibility of the sGC activity assay

**Figure S5.** sGC modulator titration

**Figure S6.** Real-time sGC titration

**Figure S7.** PDE5A activity titration

**Supplemental references**

**

Supplemental results**

**Figure S1.** Linearity of the single-label QRET assay for homogeneous monitoring of cGMP in the presence of GTP. The assay with 5 nM Eu(III)-cGMP and 4 ng of antibody (red) showed longer linear range as compared to the assay with 1 nM Eu(III)-cGMP and 2 ng of antibody (black). The observed linear ranges with 1 nM and 5 nM Eu(III)-cGMP were 0.032-20 µM and 0.032-100 µM, respectively. The presence of 0.5 mM GTP had no effect on cGMP assay linearity proving the potential for sGC activity monitoring (data not shown). In the presence of high concentration of cell lysate or mouse homogenate, the linearity is slightly shortened (data not shown). Linear range was also shortened for PDE activity assay due to the higher MT2 concentration used (data not shown). Data represent mean ± SD of three individual experiments performed in triplicates.



**Figure S2.** Eu(III)**-**cGMP binding affinity to cGMP-specific antibody. cGMP binding to antibody was estimated after conjugation of Eu(III)-label to the 2’-position of cGMP. cGMP (0-500 µM) was titrated with a fixed antibody concentration (4 ng) and five different Eu(III)**-**cGMP concentrations (0.5-10 nM). The affinity was calculated using linearized Cheng-Prusoff equation^1^. Observed EC_50_ values from each individual assays with different Eu(III)-cGMP concentrations were plotted against the used Eu(III)**-**cGMP concentration. The K_d_ value was determined from the line intersection with Y-axis. The observed K_d_ value for Eu(III)**-**cGMP binding to the used cGMP-specific antibody was 0.42 ± 0.05 µM. Data represent mean ± SD of three individual experiments performed in triplicates.

**

Figure S3.** Linearity of sGC’s cGMP-generating activity. The monitored sGC reaction is linear for up to 60 min, when 3 ng (red) and 12 ng (black) sGC are used for resting conditions and 0.5 ng sGC is used for assay with 100 µM DEA-NO (blue). The reaction was performed at 37 °C for 5, 10, 20, 40, and 60 min, stopped by detection solution (5 nM Eu(III)-cGMP, 4 ng of antibody, and 2.5 µM MT2) and TRF-signals were monitored after 10 min of incubation at room temperature. cGMP production was determined by comparing the TRF-signal monitored in sGC reaction with a cGMP standard (0-100 µM) performed parallel. Data represent mean ± SD of three individual experiments performed in triplicates.





**Figure S4.** cGMP detection platform showed good reproducibility, suitable for high throughput screening of sGC activity modulators. Assay reproducibility was monitored with 5 nM Eu(III)-cGMP and 4 ng of antibody using 24 replicate reactions for resting sGC (5 ng/well) activity (black, negative control), or sGC stimulated by 100 µM gemfibrozil (blue, positive control 1), or 10 µM DEANO (red, positive control 2). Signals were monitored 10 min (solid) or 60 min (open/dashed) after the addition of detection solution used also to stop the sGC reaction. cGMP amounts were determined based on the observed TRF-signal and cGMP standard prepared in parallel. Assays showed good reproducibility, average Z-factor with gemfibrozil and NO activated reactions were 0.74 and 0.83, respectively. Data from a single representative assay showing individual reactions (n=24) at two time point is presented. Similar results were obtained in two other experiments performed different days (data not shown).

**Figure S5.** Dose-response evaluation of sGC modulators can be performed using the QRET-based cGMP detection platform. Changes in TRF-signal showed response to sGC-dependent cGMP synthesis performed in the presence of indicated concentrations of **

**sGC activators, YC-1 (red) and riociguat (blue), and NO donor DEA NONOate (black). 5 nM Eu(III)-cGMP and 4 ng of antibody were used in detection mixture added in stop buffer. The EC_50_ values for YC-1, riociguat, and NO are 5.7 ± 1.0 µM, 0.26 ± 0.1 µM, and 0.93 ± 0.1 µM, respectively. The highest sGC activity increase was observed with DEA NONOate followed by riociguat and YC-1. Data represent mean ± SD of three individual experiments performed in triplicates.

**

Figure S6.** cGMP detection platform can be potentially used for monitoring sGC activity at real-time. Changes of TRF-signal in response to generation of cGMP by different amounts of recombinant sGC (10.2-276 ng/well) were continuously monitored in a 45 min reaction at 37 °C. Signals were recorded every 5 min during the real-time reaction. A linear reaction during the whole 45 min reaction can be observed with the lowest sGC concentration (10.2 ng/well, magenta), and the calculated sGC activity from this reaction was 41.9 ± 2.8 nmol/min/mg. When the assay was performed in the presence of NO, the calculated activities were lower than expected (data not shown). This is probably due to the loss of sGC heme, because of Triton X-100 in assay buffer. Data represent mean ± SD of three individual experiments performed in triplicates.

**

Figure S7.** PDE5A activity can be monitored using the same cGMP detection platform as with sGC. PDE5A was titrated in the presence of 5 µM (black), 10 µM (red) or 20 µM (blue) cGMP in a 60 min reaction (RT). PDE activity resulted increased TRF-signal, caused by the conversion of cGMP to GMP. During the assay, PDE activity is expected to be reduced over time due to e.g. lowered cGMP concentration, and thus the PDE specific activity should be determined in a carefully selected conditions. The detection was performed using 5 nM Eu(III)-cGMP and 4 ng of antibody similarly to sGC activity assay. A higher MT2 concentration (3.5 µM) was used to decrease the signal levels and improve the detection of small changes of cGMP levels. The QRET-based cGMP detection platform allows to detect the activity of sub-ng amounts of PDE5A. However, at these reaction conditions the S/B ratio suitable for high throughput screening of PDE inhibitors is achieved when higher than 5 µM of cGMP and at least 10 ng/well of PDE5A is used. Data represent mean ± SD of three individual experiments performed in triplicates.

**Supplemental references**

1. Newton, P., Harrison, P. & Clulow, S. A novel method for determination of the affinity of protein: protein interactions in homogeneous assays. *J. Biomol. Screening* **13**, 674-682 (2008).
